# Artificial Vector Calibration Method for Differencing Magnetic Gradient Tensor Systems

**DOI:** 10.3390/s18020361

**Published:** 2018-01-26

**Authors:** Qingzhu Li, Zhining Li, Yingtang Zhang, Gang Yin

**Affiliations:** 1 Department of Vehicle and Electrical Engineering, The Army Engineering University of PLA, Shijiazhuang 050003, China; laznlqz666@163.com (Q.L.); z.yt01@163.com (Y.Z.); gang.gang88@163.com (G.Y.); 2 High Speed Institute, China Aerodynamics Research and Development Center, Mianyang 621000, China

**Keywords:** magnetic gradient tensor system, least-squares method, vector calibration, artificial reference

## Abstract

The measurement error of the differencing (i.e., using two homogenous field sensors at a known baseline distance) magnetic gradient tensor system includes the biases, scale factors, nonorthogonality of the single magnetic sensor, and the misalignment error between the sensor arrays, all of which can severely affect the measurement accuracy. In this paper, we propose a low-cost artificial vector calibration method for the tensor system. Firstly, the error parameter linear equations are constructed based on the single-sensor’s system error model to obtain the artificial ideal vector output of the platform, with the total magnetic intensity (TMI) scalar as a reference by two nonlinear conversions, without any mathematical simplification. Secondly, the Levenberg–Marquardt algorithm is used to compute the integrated model of the 12 error parameters by nonlinear least-squares fitting method with the artificial vector output as a reference, and a total of 48 parameters of the system is estimated simultaneously. The calibrated system outputs along the reference platform-orthogonal coordinate system. The analysis results show that the artificial vector calibrated output can track the orientation fluctuations of TMI accurately, effectively avoiding the “overcalibration” problem. The accuracy of the error parameters’ estimation in the simulation is close to 100%. The experimental root-mean-square error (RMSE) of the TMI and tensor components is less than 3 nT and 20 nT/m, respectively, and the estimation of the parameters is highly robust.

## 1. Introduction

In the last decades, many kinds of magnetic gradient tensor systems based on fluxgate magnetometers or superconducting quantum interference devices (SQUID) have been developed; such systems are relatively sensitive to magnetic anomaly signals and show higher spatial resolution, therefore they have been widely used in civil and military magnetic exploration applications, such as aeronautical magnetic detection and navigation, detection of ferrous metals in the soil, searching for underground unexploded bombs, submarine investigation, and demining [1,2,3]. The magnetic gradient tensor systems comprised of magnetometers with differencing are generally composed of multiple three-axis fluxgate sensors in accordance with a certain shape combination array [4]. Many measurement factors exist that give rise to errors in measurements performed using a magnetic gradient tensor system [5,6]; because of manufacturing technology and process limitations, fluxgate sensors will always exhibit systematic errors, such as triaxial scalar output deviation and differences of sensitivity and nonorthogonality; displacement and rotation misalignment errors also arise between the different sensor axes when multiple magnetic sensors are used to arrange the tensor system. In addition, the sensor itself exhibits a core temperature coefficient and magnetic hysteresis, and hard and soft magnetic interference in the background field can also affect the measurement accuracy. The existence of these errors means that the deviation of the tensor system’s output may reach thousands of nT/m, severely affecting the measurement accuracy and necessitating the calibration of the system.

The traditional calibration approaches for a differencing magnetic gradient tensor system can be divided into the following: (1) calibration of the system error of a single magnetic sensor and (2) calibration of the misalignment error between the sensor arrays. There are two types of single magnetic sensor calibration methods, namely, vector calibration and scalar calibration. The current vector calibration method requires the use of high-precision equipment and a platform to obtain the geomagnetic field vector standard output as the reference [7], but the cost of this equipment is typically much higher than the cost of the system itself and is not suitable for practical applications. Scalar calibration is a low-cost method in which a high-precision proton magnetometer is used to measure the total magnetic intensity (TMI) scalar output for calibration reference [8]; this method ignores the actual environmental magnetic non-uniform field characteristics, resulting in overly idealized TMI calibration results, which we refer to as the “overcalibration” (OC) phenomenon. The misalignment error in current research works can be used only in vector calibration; thus, tensor system calibration is generally performed in two steps: first, the scalar method is used to calibrate the output of the individual triaxial sensors to obtain the ideal sensors’ output; then, the misalignment error is calibrated using one of the calibrated ideal sensors as a vector reference. However, this approach causes each sensor output of the system to be aligned to only one sensor and cannot use the output of the system structure’s center point as a reference. Yin et al., Pang et al., and Yu et al. have already calibrated the magnetic gradient tensor system using the two-step method [9,10,11,12,13], obtaining favorable results, and all used the scalar calibration method in the first step. In this work, we attempt to combine the advantages of both methods based on the advantages and disadvantages of vector and scalar calibrations: (1) a linear model of the sensor system error is constructed to calibrate the platform output using the scalar method and obtain the low-cost ideal vector output of the platform, and (2) the artificial platform vector output is used as a reference to integrate the 12 error parameters of the sensors in a single model, and the parameter value is estimated quickly and accurately using the least-squares nonlinear fitting method. This approach attempts to eliminate the sensor biases, scale factors, nonorthogonality error and sensor arrays’ misalignment error efficiently to improve the accuracy of parameter estimation using a low-cost procedure, providing a method and concept for the quick batch calibration of the tensor magnetic measuring instrument.

## 2. Magnetic Tensor Theory and System Construction 

The magnetic field is a vector field, and the spatial change rate of the three components in the orthogonal axes’ direction is defined as the magnetic gradient tensor [1]. Nine components exist and can be represented by the product of two vector elements as follows:

(1) $$G=\left[\begin{array}{c} \partial /\partial x\\ \partial /\partial y\\ \partial /\partial z \end{array}\right]\left[\begin{array}{ccc} B_{x} & B_{y} & B_{z} \end{array}\right]=\left[\begin{array}{ccc} \frac{\partial^{2}\varphi_{m}}{\partial x^{2}} & \frac{\partial^{2}\varphi_{m}}{\partial x\partial y} & \frac{\partial^{2}\varphi_{m}}{\partial x\partial z}\\ \frac{\partial^{2}\varphi_{m}}{\partial y\partial x} & \frac{\partial^{2}\varphi_{m}}{\partial y^{2}} & \frac{\partial^{2}\varphi_{m}}{\partial y\partial z}\\ \frac{\partial^{2}\varphi_{m}}{\partial z\partial x} & \frac{\partial^{2}\varphi_{m}}{\partial z\partial y} & \frac{\partial^{2}\varphi_{m}}{\partial z^{2}} \end{array}\right]=\left[\begin{array}{ccc} B_{xx} & B_{xy} & B_{xz}\\ B_{yx} & B_{yy} & B_{yz}\\ B_{zx} & B_{zy} & B_{zz} \end{array}\right],$$

where ***G*** is the magnetic gradient tensor matrix; *B_x_*, *B_y_*_,_ and *B_z_* are the magnetic field triaxial orthogonal components; *φ_m_* is the magnetic scalar potential; *B_ij_* (*i*, *j* = *x*, *y*, *z*) is the tensor component in the *j* direction of the *i* axis. If a magnetostatic field is present in the environment and the current is absent, according to the Maxwell equations, the magnetic field divergence and curl are equal to zero, which can be expressed as $\nabla \cdot B=\frac{\partial Bx}{\partial x}+\frac{\partial By}{\partial y}+\frac{\partial Bz}{\partial z}=0$, ∇×B=0, so that ***G*** is a symmetric matrix with a trace of zero. Thus, there are only five elements independent of each other, and these five components must be measured to obtain ***G***.

**Figure 1:**
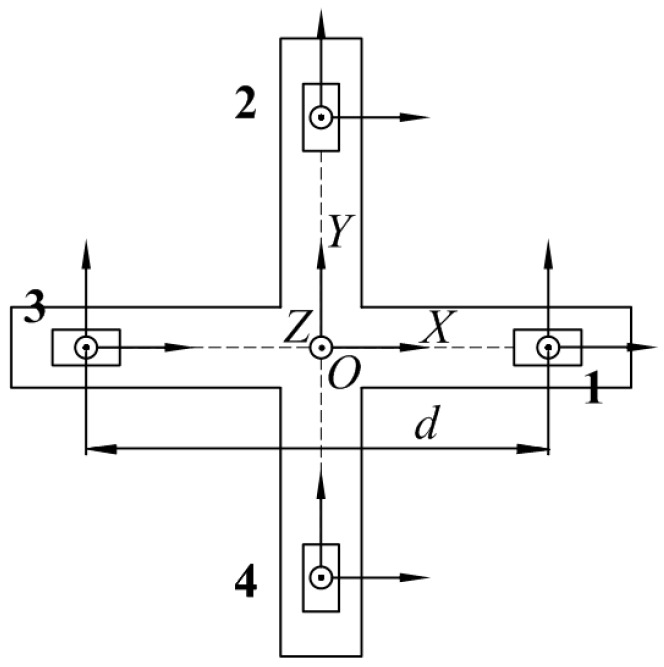
Structural design of the planar cross-magnetic gradient tensor system.

However, it is challenging to measure the gradient of the magnetic vector field in the actual measurement. Therefore, when constructing the magnetic gradient tensor measuring system, the tensor component is estimated using the difference between the measured values of the multiple magnetic sensors. Several different configurations of the magnetic gradient tensor system are analyzed in [14], and, because of the simple structure, straightforward installation, and minimal structural error, we adopt the planar cross-shaped structure to construct a magnetic gradient tensor system that consists of a planar nonmagnetic platform and four triaxis magnetic sensors. The *x* and *y* axes lie along the orthogonal baselines, and the *z* axis is chosen to make a right-handed Cartesian coordinate system. The baseline distance between two magnetometers in the same direction is *d*, as shown in Figure 1.

We use the short-distance magnetic vector difference method on the baseline to approximate the magnetic tensor gradient as *B_ij_* ≈ Δ*B_i_*/*d_j_*, where Δ*B_i_* is the component difference of the two magnetic sensors in the direction *i*, and *d_j_* is the distance between the two magnetic sensors in the direction *j*; then, the magnetic field vector ***B****_o_* at the centre point *O* and the magnetic gradient tensor matrix ***G*** can be expressed as

(2) $$\{\begin{array}{c} B_{o}=\left[\begin{array}{c} B_{xo}\\ B_{yo}\\ B_{zo} \end{array}\right]=\frac{1}{4}\left[\begin{array}{c} B_{x1}+B_{x2}+B_{x3}+B_{x4}\\ B_{y1}+B_{y2}+B_{y3}+B_{y4}\\ B_{z1}+B_{z2}+B_{z3}+B_{z4} \end{array}\right]\\ G=\frac{1}{d}\left[\begin{array}{ccc} B_{x1}-B_{x3} & B_{y1}-B_{y3} & B_{z1}-B_{z3}\\ B_{x2}-B_{x4} & B_{y2}-B_{y4} & B_{z2}-B_{z4}\\ B_{z1}-B_{z3} & B_{z2}-B_{z4} & -(B_{x1}-B_{x3})-(B_{y2}-B_{y4}) \end{array}\right] \end{array}$$

where *B_mn_* (*m = x*, *y*, *z*; *n* = 1, 2, 3, 4) represents the magnetic field component reading of the *n*th magnetic sensor in the direction *m*. The matrix shown here is not symmetric. Measurement noises and high-order gradients will create differences between the estimates of *B_xy_* and *B_yx_*. So, we average the two estimates and use a truly symmetric matrix with off-diagonal elements in the actual exploration process, and the tensor *B_xy_* and *B_yx_* components are treated separately in this paper to achieve more accurate calibration results.

## 3. Artificial Vector Calibration Method

### 3.1. System Error Parameter Model for Single Magnetic Sensors

Single magnetic sensors have a series of systematic errors, such as biases, scale factors, triaxial nonorthogonality, and temperature error. Pang et al. [15] used the least-squares support vector machine to nonlinearly compensate the temperature error of the fluxgate sensor that arises primarily from the temperature coefficient of the core material. Here, for the time being, we do not consider the temperature error because of the small difference of the working temperature and because the working time is generally sufficiently short relative to other significant system errors. Thus, we can construct a mathematical model for the error of a single sensor that contains biases, scale factors, and a nonorthogonal angle.

**Figure 2:**
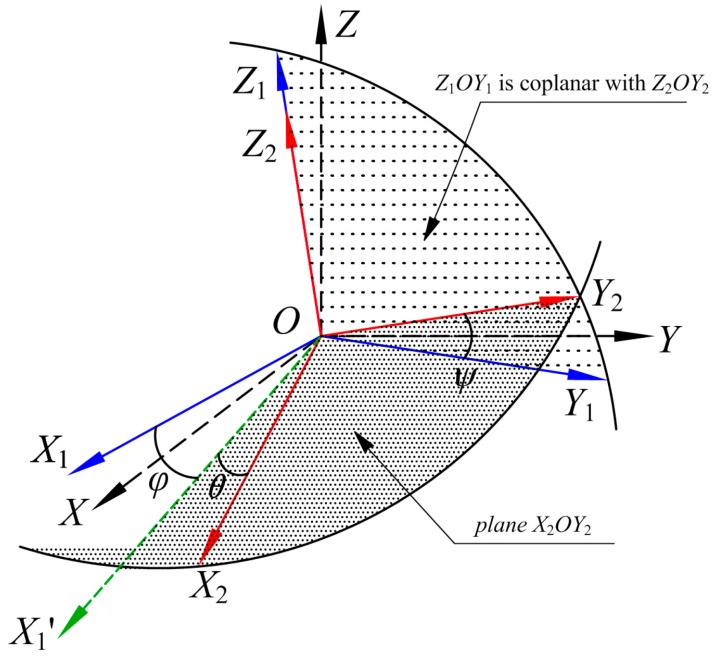
Magnetic sensor axis nonorthogonal angle.

The actual magnetic sensors are not entirely orthogonal to each other, i.e., a nonorthogonal error exists. The established nonorthogonal angles’ diagram of a single magnetic sensor triaxial coordinate system is shown in Figure 2, in which we set the actual coordinate system of the sensor as *O-X*_1_*Y*_1_*Z*_1_, the ideal orthogonal coordinate system of the sensor as *O-X*_2_*Y*_2_*Z*_2,_ and the standard reference platform frame-orthogonal coordinate system as *O-XYZ*. In Figure 2, the actual coordinate axis *OZ*_1_ of the sensor is perfectly aligned with the ideal coordinate axis *OZ*_2_, and the plane *X*_1_*OY*_1_ is coplanar with the plane *X*_2_*OY*_2_. The angle between the axis *OY*_1_ and the axis *OY*_2_ is *Ψ*, the angle between the axis *OX*_1_ and the plane *X*_2_*OY*_2_ is *φ*, and the angle between the axis *OX*_2_ and the projection *OX*_1_’ of the axis *OX*_1_ in the plane *X*_2_*OY*_2_ is *θ*. Once the nonorthogonal angles are determined, the ideal orthogonal coordinate system of the sensor is uniquely determined.

We define ***I*** = (*i_x_*, *i_y_*, *i_z_*)^T^ as the triaxis output biases and *c_i_* (*i* = *x*, *y*, *z*) as the sensitivity scale factors. Thus, we construct the parameters *θ*, *φ*, *ψ*, *c_x_*, *c_y_*, *c_z_*, *i_x_*, *i_y_*, and *i_z_*, which are the nine system error parameters of the magnetic sensor. If the actual output of the sensor is ***B***_1_ = (*B*_1*x*_, *B*_1*y*_, *B*_1*z*_)^T^ and the ideal output is ***B***_2_ = (*B*_2*x*_, *B*_2*y*_, *B*_2*z*_)^T^, then we can construct the mathematical model of the output from *O-X*_1_*Y*_1_*Z*_1_ to *O-X*_2_*Y*_2_*Z*_2_ as:

(3) $$B_{1}=\left[\begin{array}{ccc} c_{x} & & \\ & c_{y} & \\ & & c_{z} \end{array}\right]\left[\begin{array}{ccc} \cos\theta \;\cos\varphi & \sin\theta \;\cos\varphi & \sin\varphi \\ & \cos\psi & \sin\psi \\ & & 1 \end{array}\right]\times B_{2}+I=CAB_{2}+I,$$

where ***C*** and ***A*** are defined as the scale factor error matrix and the nonorthogonal error matrix, respectively. Setting *k_x_* = 1/*c_x_*cos*φ*cos*θ*, *k_y_* = 1/*c_y_*cos*ψ*, *k_z_* = 1/*c_z_*, *g* = (sin*θ*sin*ψ*cos*φ* − sin*φ*cos*ψ*)/cos*θ*cos*ψ*cos*φ*, *m* = −sin*θ*/cos*θ*, and *n* = −sin*ψ*/cos*ψ*, we obtain:

(4) $$B_{2}=\left[\begin{array}{c} B_{2x}\\ B_{2y}\\ B_{2z} \end{array}\right]=\left[\begin{array}{ccc} k_{x} & mk_{y} & gk_{z}\\ & k_{y} & nk_{z}\\ & & k_{z} \end{array}\right]\left[\begin{array}{c} B_{1x}-i_{x}\\ B_{1y}-i_{y}\\ B_{1z}-i_{z} \end{array}\right]\Rightarrow B_{2}=M(B_{1}-I).$$

Equation (4) is the error parameter calibration model of a single magnetic sensor. The parameter matrices ***M*** and ***I*** can convert the actual output ***B***_1_ of the sensor into the ideal output ***B***_2_ to complete the system error calibration process of the sensor.

### 3.2. Solving the Integrated Error Parameter Model

#### 3.2.1. Integrated Error Parameter Model

In this paper, the planar cross-magnetic gradient tensor system is arranged in pairs with four single vector triaxial magnetometers. Because of the technical limitations, we cannot guarantee the complete alignment for the magnetometer, and, after calibration of the single-sensors’ system error, the output direction of each axis changes again so that a misalignment error is present between the orthogonal coordinate systems. Pang [16] calibrated the misalignment error using one of the sensors as a reference, but this method cannot convert the output of the tensor system along the platform orthogonal system.

**Figure 3:**
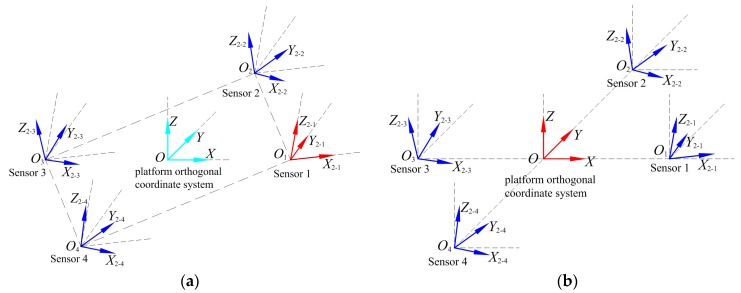
Comparison of misalignment error calibration reference system selection methods: (**a**) calibration method of Pang et al.; (**b**) calibration method of this paper.

To ensure the practicability of the calibration results, it is necessary to calibrate the misalignment errors between the ideal outputs of the sensors using the platform frame-orthogonal coordinate system as a reference, to realize an accurate measurement of the tensor system, as shown in Figure 3.

The ideal output of the sensors can be converted to the output of *O-XYZ* by rotating around three orthogonal axes. We define *α* as the roll angle of the rotation around the *X*-axis, *β* as the pitch angle of the rotation around the *Y*-axis, and *γ* as the yaw angle of the rotation around the *Z*-axis. Considering that only the roll, pitch, and yaw angles are present, we represent the roll conversion output as ***B****_α_*, the pitch conversion output as ***B****_β_*, and the yaw conversion output as ***B****_γ_*, as given by:

(5) $$\left\{ \begin{array}{l} B_{\alpha }=\left[\begin{array}{c} B_{2x}\\ B_{2y}\cos\alpha +B_{2z}\sin\alpha \\ B_{2z}\cos\alpha -B_{2y}\sin\alpha \end{array}\right]=\left[\begin{array}{ccc} 1 & & \\ & \cos\alpha & \sin\alpha \\ & -\sin\alpha & \cos\alpha \end{array}\right]\left[\begin{array}{c} B_{2x}\\ B_{2y}\\ B_{2z} \end{array}\right]=A_{\alpha }B_{2}\\ B_{\beta }=\left[\begin{array}{c} B_{2x}\cos\beta -B_{2z}\sin\beta \\ B_{2y}\\ B_{2x}\sin\alpha +B_{2z}\cos\beta \end{array}\right]=\left[\begin{array}{ccc} \cos\beta & & -\sin\beta \\ & 1 & \\ \sin\beta & & \cos\beta \end{array}\right]\left[\begin{array}{c} B_{2x}\\ B_{2y}\\ B_{2z} \end{array}\right]=A_{\beta }B_{2}\\ B_{\gamma }=\left[\begin{array}{c} B_{2x}\cos\gamma +B_{2y}\sin\gamma \\ B_{2y}\cos\gamma -B_{2x}\sin\gamma \\ B_{2z} \end{array}\right]=\left[\begin{array}{ccc} \cos\gamma & \sin\gamma & \\ -\sin\gamma & \cos\gamma & \\ & & 1 \end{array}\right]\left[\begin{array}{c} B_{2x}\\ B_{2y}\\ B_{2z} \end{array}\right]=A_{\gamma }B_{2} \end{array}\right..$$

In the above formula, ***A****_α_*, ***A****_β_*, and ***A****_γ_* are defined as the roll, pitch, and yaw rotation matrices, respectively. Thus, the output on the orthogonal coordinate of the magnetometer can be converted to the output of the reference platform.

The sequence of the three rotation matrices and the order of the output conversion route of the coordinate system are both fixed; thus, the misalignment angle calibration sequence is fixed as well. The three-axis orthogonal output of the ideal sensor is ***B***_2_ = (*B*_2*x*_, *B*_2*y*_, *B*_2*z*_)^T^, and the output being calibrated to the reference orthogonal coordinate system is ***B*** = (*B_x_*, *B_y_*, *B_z_*)^T^. Setting the order of calibration as the roll angle *α*, the pitch angle *β*, and then the yaw angle *γ*, we obtain the following output conversion:

(6) $$B=A_{\gamma }A_{\beta }A_{\alpha }B_{2}=TB_{2},$$

here, ***T*** is the rotation matrix in any spatial orientation. The ideal orthogonal output ***B***_2_ of the sensor can be converted to the output ***B*** of the reference using Equation (6), thus completing the alignment process.

According to the above derivation, the two-step model of tensor system’s error parameters can be integrated by Equations (4) and (6):

(7) $$\begin{array}{l} \left[\begin{array}{c} B_{x}\\ B_{y}\\ B_{z} \end{array}\right]=\left[\begin{array}{ccc} \cos\gamma & \sin\gamma & \\ -\sin\gamma & \cos\gamma & \\ & & 1 \end{array}\right]\left[\begin{array}{ccc} \cos\beta & & -\sin\beta \\ & 1 & \\ \sin\beta & & \cos\beta \end{array}\right]\left[\begin{array}{ccc} 1 & & \\ & \cos\alpha & \sin\alpha \\ & -\sin\alpha & \cos\alpha \end{array}\right]\left[\begin{array}{ccc} k_{x} & mk_{y} & gk_{z}\\ & k_{y} & nk_{z}\\ & & k_{z} \end{array}\right]\left[\begin{array}{c} B_{1x}-i_{x}\\ B_{1y}-i_{y}\\ B_{1z}-i_{z} \end{array}\right]\\ \Rightarrow B=TM(B_{1}-I) \end{array}.$$

#### 3.2.2. Estimation Algorithm

Equation (7) is an integrated nonlinear equation with 12 error parameters. The sensor’s actual output ***B***_1_ and the standard output ***B*** of the reference platform of *N* (*N* > 12) spatial posture points can be extended to nonlinear equations of *N* groups with different orientation:

(8) $$B=TM(B_{1}-I)\overset{extending}{\to }\left[\begin{array}{cc} B_{x_{1}} & B_{x_{2}}\\ B_{y_{1}} & B_{y_{2}}\\ B_{z_{1}} & B_{z_{2}} \end{array}\cdots \begin{array}{cc} B_{x_{N-1}} & B_{x_{{}_{N}}}\\ B_{y_{{}_{N-1}}} & B_{y_{{}_{N}}}\\ B_{z_{{}_{N-1}}} & B_{z_{{}_{N}}} \end{array}\right]=TM\times \left[\begin{array}{cc} B_{1x_{1}} & B_{1x_{2}}\\ B_{1y_{1}} & B_{1y_{2}}\\ B_{1z_{1}} & B_{1z_{2}} \end{array}\cdots \begin{array}{cc} B_{1x_{N-1}} & B_{1x_{{}_{N}}}\\ B_{1y_{{}_{N-1}}} & B_{1y_{{}_{N}}}\\ B_{1z_{{}_{N-1}}} & B_{1z_{{}_{N}}} \end{array}\right].$$

Solving the 12 specific error parameters in the nonlinear Equations (8) is crucial and can be treated as a nonlinear least-squares problem. Generally, let function ***f***: $R^{n}\to R^{m}$ be continuously differentiable with *m* ≥ *n*, and consider the nonlinear least-squares problem of finding a local minimizer of ‖***f***(*x*)‖, or, equivalently, of finding x* = argmin*_x_* {*F*(*x*)}, where

(9) $$F(x)=\frac{1}{2}\sum_{i=1}^{m}{\left(f_{i}(x)\right)}^{2}=\frac{1}{2}{\left\|f(x)\right\|}^{2}=\frac{1}{2}f{\left(x\right)}^{T}f(x),$$

which is the basic theory of the nonlinear least-squares problem [17]. Least-squares problems can be solved by general optimization methods, but we shall prefer special methods that are more efficient. We compare the merits and demerits of a variety of estimation algorithms, such as the unscented Kalman filter (UKF), genetic algorithm (GA), recursive least squares (RLS), differential evolution (DE), Gaussian–Newton (GN) iteration algorithms, and Levenberg–Marquardt (LM) algorithm, the latter being an improved form of the GN iteration method. LM is a least-squares nonlinear fitting algorithm that does not require to strictly set the initial parameters, which makes this method suitable for the actual calibration with unknown error parameters. Shawash et al., and Pang et al. [18,19] used the LM algorithm to calibrate and estimate the system parameters of instruments (such as cameras and magnetic sensors) to improve the performance of the estimation; these methods can be used as the basis for this work.

The basic idea of the LM algorithm is to use a Taylor series expansion instead of applying the nonlinear regression model in an approximate way [20]. After several iterations, the regression coefficient is corrected to approach the optimal solution of the nonlinear model continuously, and the square sum of residuals of the parameter model is minimized.

In the description of the methods in [21], we shall need formulas for derivatives of *F*: provided that ***f*** has continuous second partial derivatives, we can write its Taylor expansion as:

(10) $$f(x+h)\;=f(x)\;+J(x)h+O({\left\|h\right\|}^{2}),$$

where ‖⋅‖ denotes the 2-norm, $\left\|h\right\|=\sqrt{h_{1}^{2}+h_{2}^{2}+\cdots +h_{n}^{2}}$ and ***J*** is the Jacobian matrix.

Setting the parameter vector as ***W***(*n*) (*n =* 1, 2,..., *n*), the iterative process is represented as:

(11) W(n+1)=W(n)+ΔW(n),

where *n* is the number of iterations. According to the LM algorithm, for small ΔW we see, from the Taylor expansion (10) with inserting definition (9), that

(12) $$\left\{ \begin{array}{l} f(W+\Delta W)≃f(W)+J(W)\Delta W\\ F(W+\Delta W)≃F(W)+\Delta W^{T}J^{T}f+\frac{1}{2}\Delta W^{T}J^{T}J\Delta W=L(\Delta W) \end{array}\right.$$

(with J=J(W) and f=f(W)); it is easily seen that the gradient and the Hessian of *L* are [22]:

(13) $$L^{\prime }(\Delta W)=J^{T}f+J^{T}J\Delta W,\;L^{\prime \prime }(\Delta W)=J^{T}J.$$

Further, the matrix $L^{\prime \prime }(\Delta W)$ is independent of ΔW. It is symmetric, and if ***J*** has full rank, i.e., if the columns are linearly independent, then $L^{\prime \prime }(\Delta W)$ is also positive definite. This implies that L(ΔW) has a unique minimizer, which can be found by solving:

(14) $$\begin{array}{l} \left[J^{T}J+\mu I_{0}\right]\Delta W=-J^{T}f\\ \Rightarrow Q\Delta W=-J^{T}f \end{array},$$

where ***J*** is the Jacobian matrix of the parameter vector, ***I***_0_ is the unit matrix, *μ* is the adjustment coefficient satisfying the minimum error of the computed scalar values, and ***f*** = [***f***_1_(*W*), ***f***_2_(*W*), …, ***f****_12_*(*W*)]^T^ is the error vector of 12 estimated parameters; we define ***Q*** as a coefficient matrix, and then for all *μ* > 0, ***Q*** is positive definite, thus ensuring that Δ***W*** is in a descending channel. The nonlinear Equation (8) can be solved by invoking the lsqnonlin function in MATLAB [22] under the LM algorithm to obtain the solution vector of least-squares fitting.

### 3.3. Artificial Platform Reference Output

According to the aforementioned theory, if the platform ideal reference output ***B*** is known, then we can obtain the sensor 12 error parameters at once. However, it is challenging to measure the true magnetic field vector ***B***, and we therefore construct an artificial platform reference output as an alternative. From (2), we know that the magnetic field vector ***B****_o_* at the center point *O* is not the ideal platform output because of the lack of calibration of the actual output of each sensor. According to the description of [23], we assume that it is a truly magnetic sensor at point *O*, and then ***B****_o_* can be considered to have the same system error as a single sensor. We use the system error parameter model described in Section 3 to convert ***B****_o_* to the ideal orthogonal output ***B*,** using the measured TMI scalar as the reference by the linear method, with ***B*** serving as the artificial ideal vector output of the reference platform to achieve low-cost vector calibration. According to Equation (4), we can obtain the platform reference output transformation model as:

(15) $$\left[\begin{array}{c} B_{x}\\ B_{y}\\ B_{z} \end{array}\right]=\left[\begin{array}{ccc} k_{x} & mk_{y} & gk_{z}\\ & k_{y} & nk_{z}\\ & & k_{z} \end{array}\right]\left[\begin{array}{c} B_{xo}-i_{x}\\ B_{yo}-i_{y}\\ B_{zo}-i_{z} \end{array}\right].$$

By multiplying both sides of (15) with the transpose and defining variables *k_m,g,n_* and *i*_1,2,3_, we obtain

(16) $$\left\{ \begin{array}{l} B^{T}B={\left\|B\right\|}_{2}^{2}=k_{x}^{2}{\left(B_{xo}+k_{m}B_{yo}+k_{g}B_{zo}-i_{1}\right)}^{2}+k_{y}^{2}{\left(B_{yo}+k_{n}B_{zo}-i_{2}\right)}^{2}+k_{z}^{2}{\left(B_{zo}-i_{3}\right)}^{2}\\ \begin{array}{l} k_{m}=mk_{y}/k_{x},\;k_{g}=gk_{z}/k_{x},\;k_{n}=nk_{z}/k_{y}\\ i_{1}=i_{x}+m\frac{k_{y}}{k_{x}}i_{y}+g\frac{k_{z}}{k_{x}}i_{z}=i_{x}+k_{m}i_{y}+k_{g}i_{z}\\ i_{2}=i_{y}+n\frac{k_{z}}{k_{y}}i_{z}=i_{y}+k_{n}i_{z}\begin{array}{cc} , & \end{array}i_{3}=i_{z} \end{array}\}\Gamma_{1} \end{array}\right..$$

By merging this expression and introducing the error substitution variable *R*_1–7_ and then simplifying and expanding, we obtain the vector product form (18) as:

(17) $$\left\{ \begin{array}{l} R_{1}B_{xo}^{2}+R_{2}B_{xo}+(R_{1}k_{m}^{2}+R_{3})B_{yo}^{2}+(R_{2}k_{m}+R_{4})B_{yo}+(R_{1}k_{g}^{2}+R_{3}k_{n}^{2}+R_{5})B_{zo}^{2}+(R_{2}k_{g}+R_{4}k_{n}+R_{6})B_{zo}\\ +2R_{1}k_{m}B_{xo}B_{yo}+2R_{1}k_{g}B_{xo}B_{zo}+2(R_{1}k_{m}k_{zx}+R_{3}k_{n})B_{yo}B_{zo}+R_{7}={\left\|B\right\|}_{2}^{2}=H=B_{s}^{2}\\ \begin{array}{l} R_{1}=k_{x}^{2}\begin{array}{cc} , & \end{array}R_{2}=-2i_{1}k_{x}^{2}\begin{array}{cc} , & \end{array}R_{3}=k_{y}^{2}\begin{array}{cc} , & \end{array}R_{4}=-2i_{2}k_{y}^{2}\\ R_{5}=k_{z}^{2}\begin{array}{cc} , & \end{array}R_{6}=-2i_{3}k_{z}^{2}\begin{array}{cc} , & \end{array}R_{7}=i_{1}^{2}k_{x}^{2}+i_{2}^{2}k_{y}^{2}+i_{3}^{2}k_{z}^{2} \end{array}\}\Gamma_{2} \end{array}\right.$$

(18) $$\Rightarrow {\left[\begin{array}{c} B_{xo}\\ B_{yo}\\ B_{zo}\\ B_{xo}^{2}\\ B_{yo}^{2}\\ B_{zo}^{2}\\ B_{xo}B_{yo}\\ B_{xo}B_{zo}\\ B_{yo}B_{zo}\\ 1 \end{array}\right]}^{T}\times \left[\begin{array}{c} R_{2}\\ R_{2}k_{m}+R_{4}\\ R_{2}k_{g}+R_{4}k_{n}+R_{6}\\ R_{1}\\ R_{1}k_{m}^{2}+R_{3}\\ R_{1}k_{g}^{2}+R_{3}k_{n}^{2}+R_{5}\\ 2R_{1}k_{m}\\ 2R_{1}k_{g}\\ 2(R_{1}k_{m}k_{g}+R_{3}k_{n})\\ R_{7} \end{array}\right]=K_{1\times 10}^{T}V_{10\times 1}=H.$$

Here, ***Γ***_1_ and ***Γ***_2_ are the two nonlinear conversions, *B_s_* is the measured TMI scalar, and *H* is the square of the 2-norm of ***B***, with ***V*** defined as the substitution vector and ***K***^T^ as the signal vector. By repeating the rotary measurements of the *N* (*N* > 10) orientations of the system, ***K***^T^ can be expanded to the *N* × 10 signal matrix, and the *N* linear equations of each orientation are obtained:

(19) $$K_{N\times 10}^{T}V_{10\times 1}=H_{N\times 1}\Rightarrow V={\left(KK^{T}\right)}^{-1}KH.$$

Since the dimension of $K_{N\times 10}^{T}$ is greater than 10, the equations have no exact solution, and the estimated solution can be obtained for the ***V*** vector by the multiple least-squares estimator [24]. According to (18):

(20) $$\left\{ \begin{array}{l} R_{1}=V_{4}\begin{array}{cc} , & \end{array}R_{2}=V_{1}\begin{array}{cc} , & \end{array}R_{3}=V_{5}-V_{4}k_{m}^{2}\begin{array}{cc} , & \end{array}R_{4}=V_{2}-V_{1}k_{m}\\ R_{5}=V_{6}-V_{5}k_{n}^{2}-V_{4}(k_{g}^{2}-k_{m}^{2}k_{n}^{2})\\ R_{6}=V_{3}-V_{2}k_{n}-V_{1}(k_{g}-k_{m}k_{n})\\ k_{m}=\frac{V_{7}}{2V_{4}}\begin{array}{cc} , & \end{array}k_{g}=\frac{V_{8}}{2V_{4}}\begin{array}{cc} , & \end{array}k_{n}=\frac{V_{9}-2V_{4}k_{m}k_{g}}{2(V_{5}-V_{4}k_{m}^{2})} \end{array}\right..$$

Using the conversion ***Γ***_1_ and ***Γ***_2_, so that *k_x_* = $\sqrt{R_{1}}$, *k_y_* = $\sqrt{R_{3}}$, *k_z_* = $\sqrt{R_{5}}$, *m* = *k_m_k_x_*/*k_y_*, *g* = *k_g_k_x_*/*k_z_*, *n* = *k_n_k_y_*/*k_z_*, *i*_1_ = −*R*_2_/2*R*_1_, *i*_2_ = −*R*_4_/2*R*_3_, and *i*_3_ = −*R*_6_/2*R*_5_, we obtain the nine error parameters as follows:

(21) $$\left\{ \begin{array}{l} \theta =-\arctan k_{m}\sqrt{\frac{R_{1}}{R_{3}}}\begin{array}{cc} , & \end{array}\psi =-\arctan k_{n}\sqrt{\frac{R_{3}}{R_{5}}}\begin{array}{cc} , & \end{array}\varphi =\arctan[\cos\theta (\tan\theta \tan\psi -k_{g}\sqrt{\frac{R_{1}}{R_{5}}})]\\ c_{x}=\sqrt{\frac{1}{R_{1}}}\cos\varphi \cos\theta \begin{array}{cc} , & \end{array}c_{y}=\sqrt{\frac{1}{R_{3}}}\cos\psi \begin{array}{cc} , & \end{array}c_{z}=\sqrt{\frac{1}{R_{5}}}\\ i_{z}=-\frac{R_{6}}{2R_{5}}\begin{array}{cc} , & \end{array}i_{y}=-\frac{R_{4}}{2R_{3}}\;+k_{n}\frac{R_{6}}{2R_{5}}\begin{array}{cc} , & \end{array}i_{x}=-\frac{R_{2}}{2R_{1}}+k_{m}\frac{R_{4}}{2R_{3}}+(k_{g}-k_{m}k_{n})\frac{R_{6}}{2R_{5}} \end{array}\right..$$

The aforementioned research proves that the proposed method transforms the mathematical model into a system of linear equations by two nonlinear conversions. The above derivation process cannot be further simplified mathematically, enabling the avoidance of the deviation caused by neglecting the higher-order small quantities from the nonorthogonal angle and biases [23]. In theory, this approach can realize the completely accurate estimation of the nine error parameters of the output. Using the estimated parameters to calibrate ***B****_o_* by (15), the ideal reference platform-orthogonal output ***B*** is obtained.

## 4. Simulation 

**Figure 4:**
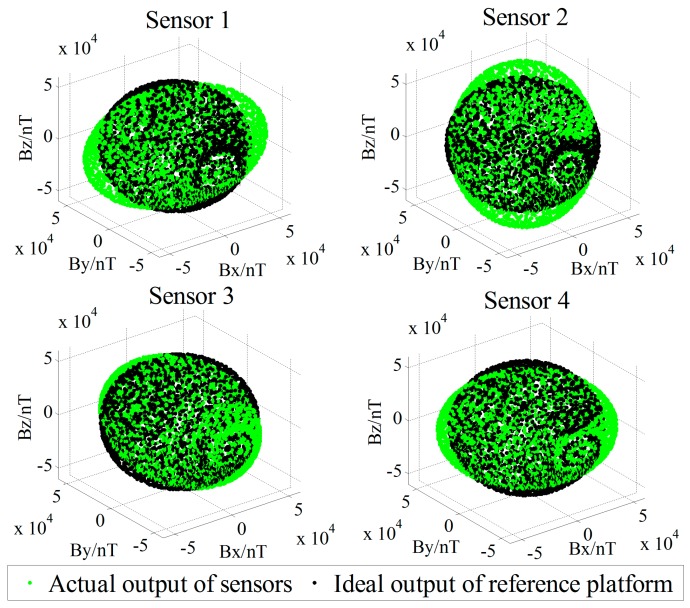
Spatial distributions of the tricomponent output in full spatial direction postures.

We attempt to verify the performance of the proposed calibration method using MATLAB simulations. We set the total field intensity as 55,000 nT, the magnetic dip as 60°, the magnetic declination as −7°, and the baseline distance of the magnetic gradient tensor system as 0.5 m. To obtain the measured data in the direction of the complete space, the simulated tensor system is rotated around the *X*, *Y*, and *Z* triorthogonal axes in turn at an interval of 20°, sampling the data 18 times per circle. Thus, there is a total of 18^3^ posture data sampled in the complete space, which is used as the ideal reference output ***B*** in postures of the full spatial direction of the standard platform. To simulate the rotational noise of the platform in the real measurement, we add Gaussian noise with a mean of 0 nT and a variance of 1 nT in the rotation process of orientations. The 48 error parameters of the four sensors are preset, and the actual tricomponent output of the sensor in the full space orientation is simulated. The tricomponents’ spatial distributions of the sensors’ actual output and the reference platform’s ideal output are contrasted in Figure 4.

**Figure 5:**
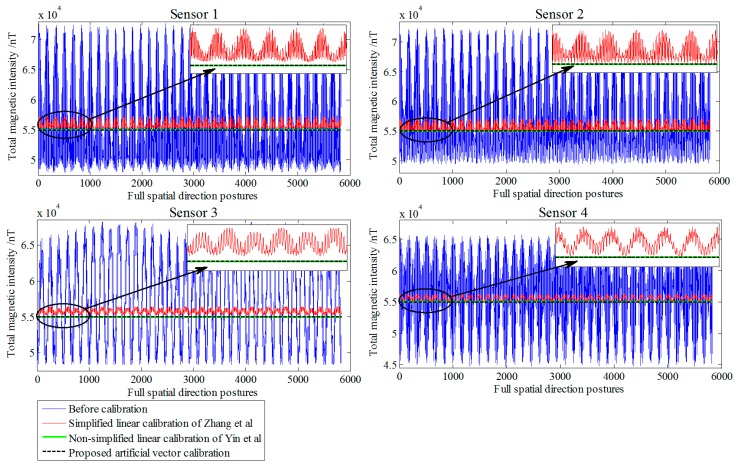
Comparison of total magnetic intensity (TMI) before and after calibration in the simulation.

**Figure 6:**
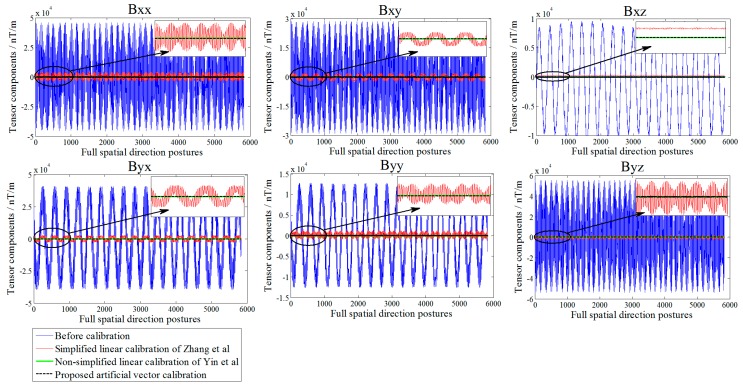
Comparison of tensor components errors before and after calibration in the simulation.

The actual tricomponent of each sensor is calibrated by the proposed method. To compare the calibration performance, we use the simplified linear calibration model of Zhang et al. [23] and the nonsimplified linear calibration model of Yin et al. [9]. Before and after the calibration, the TMI output of the sensors and the tensor components of the system center *O*-point are shown in Figure 5 and Figure 6, respectively, and the root-mean-square errors (RMSE) [25] of the sensors’ TMI are listed in Table 1, reflecting the calibration effect of the sensor system error; the RMSE values of the tensor components are listed in Table 2, reflecting the calibration effect of the misalignment error between axes, and the preset and fitting estimation parameters in simulation are listed in Table 3.

According to the simulation results, in the case of a uniform magnetic field with no hard or soft magnetic interference, when we use the simplified linear calibration of Zhang et al., the second or higher-order small quantities are neglected in the process of the single magnetometer calibration model, and the calibration deviations are brought in. Relative to the method of Zhang et al., the effect of the calibration of the nonlinear calibration method proposed in this paper is equivalent to that of the nonsimplified two-step linear calibration proposed by Yin et al., with both achieving accurate calibration in the theory of the system error in the Gaussian noise error range. However, this approach does not match the rotation order of solving the misalignment error in the step-by-step process of the linear two-step method, thus causing the estimation accuracy of the parameters to be affected by the deviation of variable conversion; this outcome is an inevitable drawback of the two-step method. The simulation results show that the parameter minimum estimation accuracy (PMEA) of the method of Yin et al. is only 86% [9], while the PMEA of the proposed nonlinear method is as high as 99.81%, enabling the achievement of the approximate lossless calibration of the error parameter model in the ideal case.

## 5. Experimental Verification 

**Figure 7:**
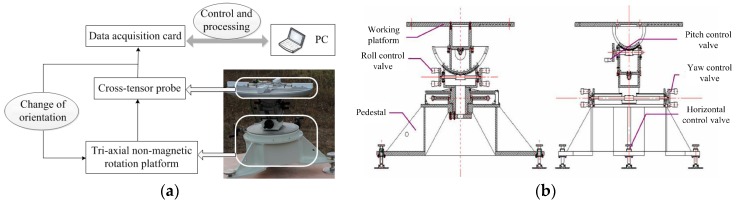
Calibration experiment of the magnetic gradient tensor system. (**a**) Experimental setup to acquire orientation data; (**b**) Design of the three-axis nonmagnetic rotation platform structure.

A planar cross-magnetic gradient tensor system is built as shown in Figure 7a, consisting of four Bartington-produced triaxial fluxgate sensors, an aluminium cross, a triaxial nonmagnetic rotation platform (the structure design is shown in Figure 7b), a data acquisition card, and a software terminal.

The material of the nonmagnetic rotation platform are aluminum and copper to avoid magnetic interference caused by operating. The main technical parameters of the platform are the following: (1) the orientation rotation range of the roll angle is 360°, and the pitch and yaw angle are ±40°; (2) the position accuracy of three Euler angles is limited to ±6’. Using Altai (Company, Beijing, China) USB2852 signal acquisition card for the data acquisition module, with 16-channel synchronous data acquisition and 16 bits of resolution, the frequency is 31 Hz−250 KHz.

Experiments in a stable environmental field with less magnetic interference were conducted in a suburb of Shijiazhuang, China. The baseline distance of the tensor system was 0.4 m, and the temperature of the working environment was 29 °C. To avoid the influence of geomagnetic diurnal variation as much as possible, the time of the experiment was chosen to be 6:00 pm. Using the scalar proton magnetometer to determine a measurement point with a comparatively more stable uniform magnetic field, the average TMI scale value *B*_s_ of the tensor system in the rotating space was 53,902.87 nT, and the range of fluctuation was ±10 nT for different orientations.

**Figure 8:**
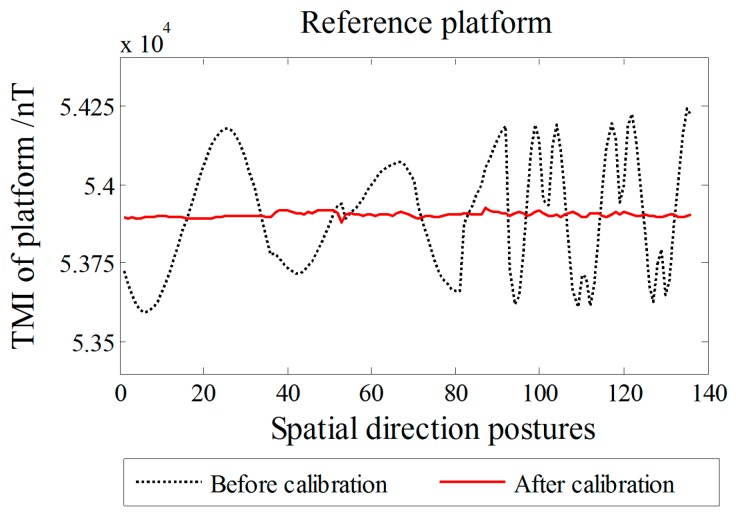
Artificial reference platform output before and after calibration.

The experimental process was divided into two parts, the first being conducted around the *Z*-axis of the platform for a standard measurement, and the second being a random orientation measurement, i.e., a random rotation of the nonmagnetic platform for arbitrary space orientation measurement point sampling. Standard measurements were sampled once per 10°, and a total of 36 samples were taken around the *Z*-axis per circle. Random sampling was performed a total of 100 times, thus increasing the amount of data to avoid accidental results and ensure the effect and adaptable performance of the calibration. A total of 136 sets of spatial direction posture data were sampled, each of which contains the magnetic field tricomponent output of each orientation for four sensors. According to (2), we obtain an average of tricomponents of the four sensors’ output to ***B****_o_*, and we use *B_s_* as the reference to calibrate ***B****_o_* by the linear method of Section 5, to construct the artificial-reference-platform ideal output ***B*** = (*B_x_*, *B_y_*, *B_z_*)*^T^* of the cross tensor system center *O*-point. A comparison of the reference platform outputs before and after calibration is shown in Figure 8. The RMSE of the TMI of the artificial platform output ***B*** is 7.4 nT after the calibration, which is within the range of the TMI orientation fluctuation, proving the validity of ***B***.

**Figure 9:**
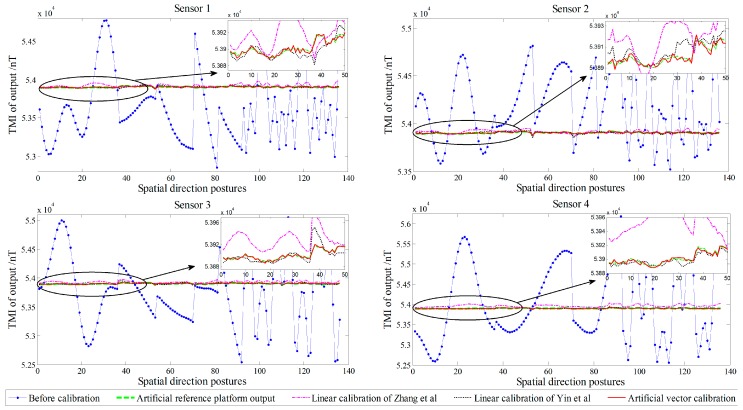
Comparison of TMI before and after calibration in the experiment.

**Figure 10:**
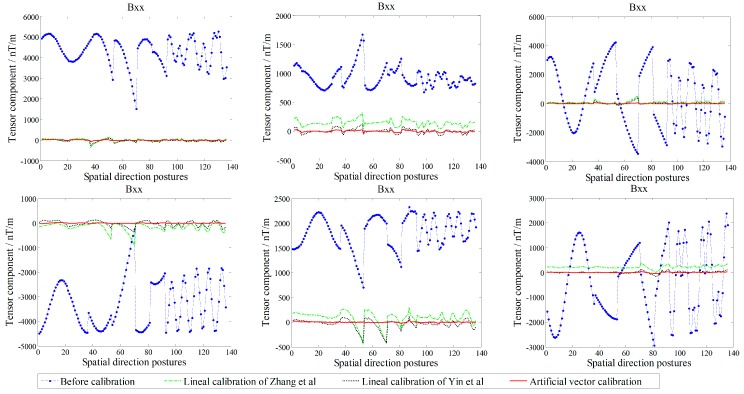
Comparison of tensor components before and after calibration in the experiment.

**Figure 11:**
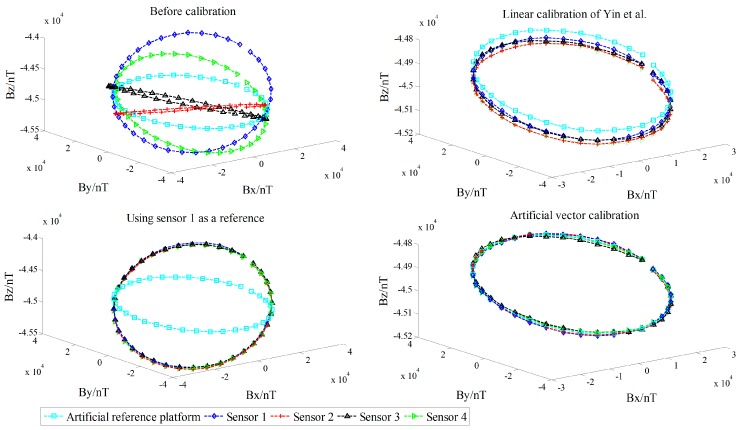
Spatial distributions of tricomponents before and after calibration of the first 36 orientations.

All data were calculated using the linear calibration method of Yin et al., Zhang et al., and the proposed artificial vector calibration method. We calibrated the output of the four sensors after obtaining a total of 48 estimated error parameters. The calibration effect of TMI for each sensor is shown in Figure 9, with the corresponding RMSE presented in Table 4. Since the ambient magnetic field is a nonuniform field, and there are diurnal and environmental magnetic interferences, the true field intensity of the geomagnetic field is not constant but fluctuating; therefore, along with the orientation change process, the real geomagnetic total field can be represented by 136 sets of the reference platform output ***B*** and is used as a reference in Figure 9. The six independent tensor components of the system before and after calibration are shown in Figure 10, with the corresponding RMSE values listed in Table 5. For comparison, Figure 11 shows the linear calibration of Yin et al. [9], the aligning idea of Pang et al. [16] when using one of the sensors as a reference, and the spatial distributions of the sensor’s magnetic field tricomponents before and after calibration of the first 36 orientations of standard measurements rotating around the *Z*-axis. The total field output RMSE [25] is given by:

(22) $$E_{\mathrm{RMS}}=\sqrt{(\sum_{i=1}^{N}{\left(Bc_{i}-B_{i}\right)}^{2})/N},$$

where *B_i_* is the reference platform output of the *i*th posture point, $Bc_{i}$ is the calibration output of the *i*th posture point, and *N* is the number of orientations. We can see that the accuracy of the simplified linear calibration of Zhang et al. is slightly worse. The results of the experimental comparison show that we must use *B*_s_ as the reference for the two-step linear calibration of Yin et al. because of the calculation requirements; however, this approach ignores the fluctuation along with the orientation change of the magnetic field intensity in the actual environment, resulting in the OC phenomenon, so that the coaxial output between the sensors and the reference platform is affected after calibration. However, this result is not reflected in the simulation process described in Section 6 for the OC phenomenon because of the set ideal case. By contrast, the sensor can fit the reference platform output accurately with the proposed vector calibration method, the fluctuant tracking performance is improved, the RMSE of TMI is reduced to less than 1 nT, and the accurate calibration is achieved in the abovementioned average TMI scale fluctuation range of vector calibration. For the misalignment error, the aligning idea of Pang et al. exhibits a satisfactory coaxiality after calibration, but it cannot be output along the reference platform, making this approach impractical, while the performance of the linear method of Yin et al. is affected by the output coincidence degree because of the OC phenomenon for a single sensor. The RMSE of the tensor components is substantially reduced by the proposed method, and the tricomponent spatial distributions of the sensors magnetic field are more coaxial and show a stronger degree of coincidence with the reference platform output.

To verify the robust performance of the 48 error parameters estimated by this method, we reselect the measurement point. The obtained results show that the reproduction degree of each parameter after two estimation iterations is higher than 95% in Table 6, indicating that the calibration results are stable and reliable.

## 6. Conclusions

This paper proposes an artificial vector calibration method for differencing magnetic gradient tensor systems. We use the linear calibration method to construct the artificial ideal platform output as the reference vector. The Levenberg–Marquardt algorithm is used to realize the least-squares fitting of nonlinear equations by establishing an integrated nonlinear mathematical model of the single-sensor system error of biases, scale factors, nonorthogonal angles, and the measurement error of the sensor arrays. A total of 48 parameters of the four sensors is estimated simultaneously, providing the concept and method for the accurate calibration of aeronautical, underwater, and surface tensor magnetic measuring instruments. As a result of using the multiorientation single-sensor vector output calibration, the method is suitable for any triaxial magnetic sensor or accelerometer array combination of the magnetic field, and the gravity field tensor system with an accurate and efficient parameter estimation can achieve batch and rapid calibration of tensor measurement instruments, contributing to the scientific literature and the commercial value. Relative to the calibration methods of Zhang et al., Yin et al., and Pang et al., in the ideal case of a uniform magnetic field, the accuracy of parameter estimation with the nonlinear integrated calibration is close to 100% in simulation, and a lossless calibration is realized. As a result of the lack of the integrated parameter model, the defects of the fixed solving sequence and distortion of conversion in the two-step method are inevitable, while, experimentally, the ability of tracking the calibration for the magnetic field output fluctuates, following the orientation change with the proposed method, effectively avoiding the OC problem of the linear calibration, which must set the total field intensity to a constant value to solve the linear equations.

In this paper, the idea and method of a low-cost vector calibration for tensor systems are provided, and the estimation of the parameters is comparatively accurate. However, we have not considered the influence of the sensor temperature coefficient, nonlinearity, or hard or soft magnetic interference on the accuracy of the tensor system, and the algorithm has a strong dependence on vector output based on the standard reference platform. In the future, multiorientation magnetic field vector measurement data can be used as the tensor system reference outputs with a more sensitive and high-frame magnetometer to improve the calibration accuracy and reliability.

## Figures and Tables

**Table 1:** Comparison of root-mean-square errors (RMSE) of TMI before and after calibration using the simulation.

Process	Sensor 1 (nT)	Sensor 2 (nT)	Sensor 3 (nT)	Sensor 4 (nT)
Before calibration	6685.23	6638.08	6548.99	4951.23
“Zhang” calibration	1003.92	927.04	827.88	690.39
“Yin” calibration	0.5835	0.5767	0.5706	0.5703
My calibration	0.5837	0.5768	0.5710	0.5704

**Table 2:** Comparison of RMSE of tensor components before and after calibration using the simulation.

Process	Bxx (nT/m)	Bxy (nT/m)	Bxz (nT/m)	Byx (nT/m)	Byy (nT/m)	Byz (nT/m)
Before calibration	24,411.4	15,523.2	6791.5	25,222.8	8000.2	29,176.6
“Zhang” calibration	2166.6	1243.5	286.5	1615.5	595.0	970.3
“Yin” calibration	1.7692	1.7361	1.7594	1.7945	1.6692	1.7999
My calibration	1.6287	1.6102	1.6221	1.6313	1.6285	1.6282

**Table 3:** Preset and estimated system error parameters in the simulation.

Errors	Preset Parameters				Estimated Parameters				PMEA %
	Sensor 1	Sensor 2	Sensor 3	Sensor 4	Sensor 1	Sensor 2	Sensor 3	Sensor 4	
θ/°(rad)	−2.46	−3.88	1.69	−2.62	−2.458 (−0.0429)	−3.879 (−0.0677)	1.690 (0.0295)	−2.618 (−0.0457)	99.92
φ/°(rad)	3.53	1.73	1.44	−1.45	3.529 (0.0616)	1.730 (0.0302)	1.438 (0.0251)	−1.450 (−0.0253)	99.86
ψ/°(rad)	1.14	1.55	3.62	2.31	1.140 (0.0199)	1.553 (0.0271)	3.621 (0.0632)	2.309 (0.0403)	99.81
$c_{x}$	1.312	0.925	0.897	1.185	1.3120	0.9250	0.8970	1.1850	100.00
$c_{y}$	0.915	0.943	1.231	1.044	0.9150	0.9430	1.2310	1.0440	100.00
$c_{z}$	0.881	1.315	0.888	0.818	0.8810	1.3150	0.8880	0.8180	100.00
$i_{x}$/nT	351	131	201	218	351.0153	131.0000	201.0034	217.9913	100.00
$i_{y}$/nT	111	−294	−335	−334	110.9985	−293.9941	−334.9951	−334.0055	100.00
$i_{z}$/nT	−208	217	99	−251	−207.9969	217.0019	98.9976	−250.9985	100.00
α/°(rad)	−2.93	2.64	2.92	1.64	−2.928 (−0.0517)	2.641 (0.0461)	2.922 (0.0510)	1.639 (0.0286)	99.93
β/°(rad)	1.75	3.19	1.88	0.89	1.748 (0.0305)	3.191 (0.0557)	1.879 (0.0328)	0.888 (0.0155)	99.89
γ/°(rad)	2.28	0.82	−3.05	−2.54	2.280 (0.0398)	0.819 (0.0143)	−3.048 (−0.0532)	−2.538 (−0.0443)	99.92

**Table 4:** RMSE of TMI before and after calibration in the experiment.

Process	Sensor 1 (nT)	Sensor 2 (nT)	Sensor 3 (nT)	Sensor 4 (nT)
Before calibration	536.10	482.39	621.70	1028.26
“Zhang” calibration	26.1744	18.5793	30.4573	68.3018
“Yin” calibration	7.9052	9.2214	9.4605	6.4840
My calibration	2.3857	2.4509	1.7233	2.1041

**Table 5:** RMSE of tensor components before and after calibration in the experiment.

Process	Bxx (nT/m)	Bxy (nT/m)	Bxz (nT/m)	Byx (nT/m)	Byy (nT/m)	Byz (nT/m)
Before calibration	4365.18	958.38	2211.11	3425.93	1849.67	1474.37
“Zhang” calibration	73.3097	156.994	122.685	223.926	159.994	226.273
“Yin” calibration	58.6183	41.9891	77.3008	119.558	104.468	46.3363
My calibration	13.3575	13.4651	15.3108	10.3117	7.9620	13.9699

**Table 6:** Estimation error parameters of different measurement data after the calibration experiments.

Errors	Sensor 1		Sensor 2		Sensor 3		Sensor 4	
	Data 1	Data 2	Data 1	Data 2	Data 1	Data 2	Data 1	Data 2
θ**/°**	−0.252	−0.257	0.705	0.700	−0.109	−0.111	0.819	0.823
φ**/°**	−3.478	−3.477	3.071	3.086	−3.146	−3.150	3.174	3.167
ψ**/°**	0.888	0.886	0.865	0.873	−1.793	−1.797	2.498	2.481
$c_{x}$	0.999	0.999	1.008	1.007	0.996	0.995	1.006	1.007
$c_{y}$	1.004	1.005	1.001	1.000	1.004	1.003	1.004	1.004
$c_{z}$	0.994	0.993	0.999	0.998	0.997	0.997	0.996	0.995
$i_{x}$/nT	361.0	363.4	382.9	387.1	−105.9	−103.6	−310.3	−303.8
$i_{y}$/nT	−244.7	−248.4	196.1	201.3	218.3	220.2	−243.3	−238.7
$i_{z}$/nT	−47.7	−51.5	−187.2	−190.9	−131.7	−136.8	−83.2	−81.1
α/°	0.676	0.677	−0.911	−0.907	−0.481	−0.485	0.705	0.706
β/°	−0.871	−0.868	0.241	0.246	0.768	0.767	−0.155	−0.152
γ/°	−2.154	−2.152	1.633	1.641	−1.369	−1.379	1.885	1.873
